# Adherence to the dietary approaches to stop hypertension trial (DASH) diet is inversely associated with incidence of insulin resistance in adults: the Tehran lipid and glucose study

**DOI:** 10.3164/jcbn.16-95

**Published:** 2017-08-11

**Authors:** Saeed Esfandiari, Zahra Bahadoran, Parvin Mirmiran, Maryam Tohidi, Fereidoun Azizi

**Affiliations:** 1 Nutrition and Endocrine Research Center, Research Institute for Endocrine Sciences, Shahid Beheshti University of Medical Sciences, 19395-4763 (P.O. Box), No. 24., Shahid Erabi St., Yeman St., Velenjak, Tehran, Iran; 2 Student Research Committee, Research Institute for Endocrine Sciences, Shahid Beheshti University of Medical Sciences, 19395-4763 (P.O. Box), No. 24., Shahid Erabi St., Yeman St., Velenjak, Tehran, Iran; 3 Prevention of Metabolic Disorders Research Center, Research Institute for Endocrine Sciences, Shahid Beheshti University of Medical Sciences, 19395-4763 (P.O. Box), No. 24., Shahid Erabi St., Yeman St., Velenjak, Tehran, Iran; 4 Endocrine Research Center, Research Institute for Endocrine Sciences, Shahid Beheshti University of Medical Sciences, 19395-4763 (P.O. Box), No. 24., Shahid Erabi St., Yeman St., Velenjak, Tehran, Iran

**Keywords:** dietary approaches to stop hypertension, insulin resistance, insulin

## Abstract

Beneficial effects of Dietary Approaches to Stop Hypertension trial (DASH) diet on features of metabolic syndrome have been indicated in clinical studies. In this study, we aimed to assess possible association of DASH diet score and the risk of insulin resistance in an Iranian population. In this prospective cohort study, 927 adult men and women, were recruited. Fasting serum insulin and glucose were measured at baseline and again after 3 years. Usual dietary intakes were measured using a validated 168 item semi-quantitative food frequency questionnaire and DASH score was calculated. Multivariate logistic regression models were used to estimate the occurrence of the insulin resistance across tertiles of DASH diet. To investigate possible superiority of DASH score over other scoring system, we also assessed the association of healthy eating index and Mediterranean diet score with the risk of insulin resistance. Mean age of the participants was 40.34 ± 12.14 years old. The incidence rate of insulin resistance was 12.8%. Participants with higher DASH score had also higher intakes of potassium, calcium, magnesium, fiber, and lower intakes of cholesterol (*p*<0.05). After 3-years of follow-up, a significant negative association was observed between DASH score and the risk insulin resistance in the highest compared to the lowest tertile (OR = 0.39, 95% CI = 0.20–0.76, *p* for trend = 0.007). There was no significant association between healthy eating index and Mediterranean diet score with the incidence of insulin resistance. In conclusion, adherence to the DASH dietary pattern may be associated with a lower risk of insulin resistance and its related metabolic outcomes.

## Introduction

Insulin resistance (IR), defined as a state of reduced responsiveness to normal circulating levels of insulin, plays a major role in the development of type 2 diabetes and its related metabolic disorders.^(1)^ The effectiveness of lifestyle modification approaches in prevention and management of cardiometabolic disorders have been well recognized and there has been growing interest regarding the potential effects of dietary factors on the development of type 2 diabetes and IR.^(2)^ Previously, we reported that food patterns and some dietary factors including dietary acid load, dietary insulin index, as well as consumption of phytochemical-rich foods have a considerable contribution in the development of IR and β-cell dysfunction.^(3–6)^

The Dietary Approaches to Stop Hypertension (DASH) diet, which encourages the high intake of whole grain, fruits, vegetables, and low-fat dairy products combined with limit consumption of saturated fat, red meats, sweets, and sugar-containing beverages, was originally developed to prevent hypertension.^(7,8)^ In comparison with usual diets the DASH diet provides lower amounts of total fat, saturated fat, and dietary cholesterol, whereas providing higher amounts of potassium, calcium, magnesium, fiber, and protein. Furthermore, DASH diet is now recommended as an ideal eating dietary pattern for all adults.^(9,10)^ High fiber, antioxidant components, unsaturated fatty acids, and low-fat dairy in DASH diet can also improve insulin homeostasis and glucose tolerance.^(11)^

Although this is well established that DASH diet can reduce blood pressure, its influence on insulin homeostasis still less known. Observational data from the Insulin Resistance Atherosclerosis Study suggested that the DASH diet may prevent the development of diabetes in some individuals,^(12)^ however findings from clinical investigations are still inconsistent. Beneficial properties of DASH diet on IR did not confirmed in some clinical trials,^(13–16)^ whereas improvement of homeostasis model assessment of insulin resistance (HOMA-IR) was observed following adherence to DASH diet polycystic ovary syndrome (PCOS) and pregnant women with gestational diabetes.^(17,18)^ Adherence to DASH diet combined with a training program improved insulin sensitivity in patients with hypertension and overweight.^(19)^

Although adherence to the DASH dietary pattern indicates some potential properties against development of diabetes and metabolic syndrome, persistent challenges remain for beneficial effect of consumption DASH diet with IR Existing data suggest that potential effect of DASH diet on IR is expected in the context of weight loss program, or in some pathologic conditions such as PCOS or gestational diabetes mellitus.^(11,17,18)^

To address this gap and clarify probable effect of the DASH diet on incidence of IR over the time, therefore, we conducted the current investigation in a large representative Iranian population participated in the Tehran Lipid and Glucose Study (TLGS) during 2006–2011. We also assessed the association of healthy eating index (HEI) and Mediterranean diet score with the risk of IR.

## Methods

### Study population

Tehran Lipid and Glucose Study (TLGS) is a community-based prospective study designed to investigate and prevent non-communicable diseases, in a representative sample in district 13 of Tehran, the capital city of Iran.^(19)^ First Adult men and women with complete data (demographics, anthropometrics, biochemical and dietary data) that participated in the third (2006–2008) and fourth (2009–2011) phases of TLGS study were included in study. Then Participants who had unexplained energy intake (<800 kcal/day or >4,200 kcal/day) or were on specific diets (*n* = 262), had no follow-up information on anthropometrics and biochemical measurements at the second examination (2009–2011), excluded from study. After exclusion of the participants with IR at baseline, finally, data of 927 participants was included in the analysis. The mean duration of the follow-up was approximately 3 years.

Written informed consents were obtained from all participants and the study protocol was reviewed and approved by the ethics research council of the Research Institute for Endocrine Sciences, Shahid Beheshti University of Medical Sciences.

### Demographic and anthropometric measures

 Demographics, anthropometrics, and biochemical measures were obtained at baseline and after 3-years follow-up. Trained interviewers team collected information using local validated questionnaires. Smoking status was obtained during face-to-face interviews. Physical activity levels measured according to the frequency and time spent on light, moderate, high and very high intensity activities, according to the list of common activities of daily life over the past year. Physical activity levels were expressed as metabolic equivalent hours per week (METs h/week). Digital scale was used to obtain weight measurement with nearest of 100 g, while the subjects were removed their bulky clothe and shoes. Standing Height was measured to the nearest 0.5 cm with using a measuring tape. Body mass index (kg/m^2^) was calculated as weight (kg) divided by the square of height (m). Waist circumference (WC) was measured to the nearest 0.1 cm (at anatomical landmarks), at the widest portion, over light clothing, using a soft, tape meter, without any pressure to the body.

### Biochemical measures

Fasting blood samples were taken at baseline and after 3-years of follow-up from all study participants in the early morning and after 12–14 h fast. Fasting serum insulin was quantified by the electrochemiluminescence immunoasaay (ECLIA), using Roche Diagnostics kits and the Roche/Hitachi Cobas e-411 analyzer (GmbH, Mannheim, Germany). The intra- and inter-assay coefficients of variation for insulin were 1.2 and 3.5%, respectively. Fasting plasma glucose was measured by the enzymatic colorimetric method using glucose oxidase. Inter- and intra- assay coefficient of variation of glucose assays was <5%. IR was assessed using the HOMA-IR as follows: Fasting insulin (µU/ml) × fasting glucose (mM)/22.5; this index has been developed as a simple, inexpensive, and validated alternative tools for assessment of IR in epidemiological studies.^(20–22)^ In the present study, IR was defined as HOMA-IR ≥3.2.^(23)^

### Dietary assessment

Local validated 168-item food frequency questionnaire (FFQ) was used to assess typical food intake at first examination over the previous year. Trained dietitians asked participants to designate their intake frequency for each food item, consumed during the past year on a daily, weekly, or monthly basis. Portion sizes of consumed foods reported in household scales were then converted to grams. Energy and nutrient content of foods and beverages were obtained with using US Department of Agriculture Food Composition Table (FCT) because of incomplete Iranian FCT and limited data on nutrient content of raw foods and beverages.^(24)^ Finally, dietary intakes of participants including dietary energy and energy density, macronutrients, micronutrients, and food groups were determined.

### Calculation of diet quality scores

We constructed the DASH score based on 8 components: high intake of fruits, vegetables, nuts and legumes, low-fat dairy products, and whole grains and low intake of sodium, sweetened beverages, and red and processed meats. For each component, individuals were classified into quintiles according to their intake ranking. For high recommended components: fruits, vegetables, nuts and legumes, low-fat dairy products, and whole grains, quintile 1 assigned 1 point and quintile 5 assigned 5 point but for low recommended components: sodium, sweetened beverages, and red and processed meats, quintile 1 assigned 5 point and quintile 5 assigned 1 point. Sum of 8 component scores indicated total DASH score that ranged from 8 to 40. The HEI is structured based on 12 dietary components and divided in tow main categories; adequacy and moderation. The Adequacy contains 9 components as: total fruit, whole fruit, total vegetables, greens and beans, whole grains, dairy, total protein foods, seafood and plant proteins and fatty acid [(PUFAs + MUFAs)/SFAs]. The moderation contains 3 components as: refined grains, sodium and empty calories. The HEI diets are scored on a density basis (per 1,000 kcal) for whole fruits, whole fruits, all vegetables, beans, peas, dairy products, meat, seafoods, plant proteins, whole grains, refined grains and sodium. The maximum possible score on the index is 100 (optimal diet) and gives equal weight to all 10 components (0–10 each) while taking minimal account of age and sex differences. Point values are calculated proportionally for consumed quantities which fall between the maximum and minimum levels of consumption.^(25)^ Adherence to the Mediterranean eating plan was assessed with an index variable that was composed of the 9 Mediterranean food groups.^(26)^ For each food group, a value of 0 or 1 was assigned with the use of the sex-specific median as the cutoff. Mediterranean diet was mudified for removing alcohol because of consumption prohibition. For beneficial components (vegetables, legumes, fruits and nuts, cereal and fish), value of 1 was assigned as consumption above the median and value of 0 was assigned as consumption below the median. For detrimental components (meat, poultry and whole dairy products) value of 0 was assigned as consumption above the median and value of 1 was assigned as consumption below the median. Maximum point is 8 and minimum is 0. In the current study, for make Mediterranean diet scoring comparable with DASH scoring system, the original Mediterranean diet score was modified as follow: each Mediterranean diet components scored 1 to 5 based quintile of intake. Reverse scoring used for meat, poultry and whole dairy products. Maximum point for this scoring system is 40 and minimum is 8.

### Statistical analysis

The Kolmogrov-Smirnov test was applied to ensure the normal distribution of variables. Log transformation was performed for non-normally distributed variables. Dietary DASH, Mediterranean and HEI components were adjusted for total energy intake, based on the residuals methods.^(27)^ The characteristics of the study population were compared across tertile categories of dietary DASH, Mediterranean and HEI score, using the analysis of variance or chi-square test. A univariate analyses was performed for potential confounding variables; variables with P_E_<0.2 in the univariate analyses were selected for the final multivariable models; P_E_ (*p* value for entry) determines which variables should be included in the multivariable model. To estimate the risk of IR across tertiles of dietary DASH, Mediterranean and HEI score, multiple regression models were used. The potential confounding variables adjusted in the models were sex, age at baseline (year, continuous), waist circumference (cm), smoking status (yes/no), physical activity (MET-h/week, continuous), anti-diabetic medications (yes/no), baseline serum insulin and HOMA-IR and total energy intakes (kcal/day). Because we explicitly wanted to evaluate the contribution of demographic and lifestyle variables to the associations under study, we present this model second. A linear trend test was performed considering each ordinal score variable as a continuous variable in the model. To assess the overall trends of odds ratios of IR across tertiles of dietary DASH, Mediterranean and HEI score, the median of each tertile was used as a continuous variable in the logistic regression models.

All statistical analyses were performed using SPSS (ver. 16.0; Chicago, IL), and results were considered significant at *p* values <0.05.

## Results

Mean age of participants was 40.34 ± 12.14 years, at baseline. The incidence rate of IR was 12.8% after 3-years. The Characteristics of the study population across tertiles of DASH diet score are shown in Table 1. Subjects with the highest DASH score were more likely to be older and women (*p*<0.05). The Characteristics of the study population across tertiles of Mediterranean and HEI score are shown in Table 2. Dietary intakes of the participants across tertiles of dietary DASH score are shown in Table 3. Total energy intake was significantly higher in the highest compared to the lowest tertile categories of DASH (2,298 vs 2,390 kcal/day, *p*<0.05). Participants with higher DASH score had also higher intakes of potassium, calcium, magnesium, fiber, and lower intakes of cholesterol (*p*<0.05). Dietary intakes of vegetables, fruits, low fat dairy products, whole grains, legumes and nuts were higher in the highest compared to the lowest tertile of DASH score whereas red and process meat and sweetened beverages were lower in the participants with higher DASH score (*p*<0.05). Dietary intakes of the participants across tertiles of Mediterranean and HEI score are shown in supplementary Table 4, 5 and 6.

The risk of IR (odds and 95% CI) in each tertile of DASH score as well as Mediterranean and HEI score are shown in Table 7. After 3-years of follow-up, we found a significant negative association between DASH dietary score and the risk IR in the highest compared to the lowest tertile, in the age- and sex-adjusted model (OR = 0.52, 95% CI = 0.31–0.89, *p* for trend = 0.01); in the model 3, additionally adjusted for waist circumference, the risk IR decreased by 54% in the participants had DASH score ≥24 (OR = 0.46, 95% CI = 0.26–0.80, *p* for trend = 0.007). In the model 4, adjusted for further potential confounders including smoking and physical activity, the risk of IR significantly decreased across increasing dietary DASH score (OR = 0.87, 95% CI = 0.52–1.45, and OR = 0.49, 95% CI = 0.26–0.90, in the second and third tertile, respectively, *p* for trend = 0.02). In the final model, after additional adjustment of energy intake, baseline values of HOMA-IR and serum insulin, and anti-diabetic medications as well, protective effect of higher adherence to DASH diet against development of IR remained still significant (OR = 0.39, 95% CI = 0.20–0.76, *p* for trend = 0.007). There was no significant association between HEI and Mediterranean diet score with the incidence of IR.

## Discussion

To our knowledge, the present study is the first to address the relation of adherence to the DASH diet and IR in the framework of longitudinal population-based study. Although there was no significant difference in insulin levels and IR index across tertiles of DASH score, our multivariate models implied that adherence of DASH diet pattern may decrease the development of IR. Lack of significant correlation between DASH score and insulin levels and HOMA-IR and in contrast, significant association between DASH and risk of IR after 3 years of follow-up, may be due to potential confounding variables adjusted in the multivariate models. Other scores including HEI and Mediterranean diet score had no significant association with the risk of IR. Our findings indicated superiority of DASH score over the other scoring system (HEI and Mediterranean diet score) in prediction of IR in our population.

Findings of the current study are in line with our previous findings showed that healthy pattern typified by frequent consumption of fresh and dried fruits, low-fat dairy, vegetable oils, nuts and seeds reduced both fating and postprandial glucose and insulin levels as well as IR index and decreased hyperinsulinemia.^(4)^ Our previous study also showed that higher adherence to DASH diet was related to lower incidence of metabolic syndrome and it components including hypertension, high fasting plasma glucose, and abdominal obesity.^(28)^

The effects of DASH diet on HOMA-IR have previously been studied in participants with the metabolic syndrome, type 2 diabetes, PCOS, gestational diabetes, abdominal obesity and pre-hypertensive patients. Some clinical studies have found that DASH diet had beneficial effect on management of IR and disglycemia, whereas some have found no similar effect. It should be noted that the beneficial effects of DASH dietary pattern on IR mainly observed among participants with metabolic disorders.^(18,29–31)^ Adherence to the DASH eating pattern for 8 weeks in overweight and obese women with PCOS resulted in improved insulin sensitivity.^(17)^ In another clinical trial, adherence to the DASH diet for 4 week in pregnant women with gestational diabetes, had beneficial effects on serum insulin levels and HOMA-IR.^(18)^ Decreased IR was also observed following a 20-week adherence to DASH diet in pre-hypertensive individuals.^(30)^ Similar findings have also been reported in secondary analysis of clinical trials, the established and established-plus-DASH interventions both led to significant decreases in fasting insulin levels and in the homeostasis model index of IR,^(14)^ and in postmenopausal women with the metabolic syndrome after 8 weeks.^(32)^

In contrast, lack of beneficial effect was reported on insulin sensitivity following 6 months adherence to of DASH dietary pattern, in both subjects with and without metabolic syndrome.^(14)^ In another clinical study, the insulin sensitivity index remain unchanged following adherence to DASH diet, over the period of the 6-month intervention.^(33)^

Several mechanisms may be proposed to explain protective effects of DASH diet against IR. Studies have shown that higher intakes of whole grains, as important components of DASH diet, could increase insulin sensitivity; fermentation of indigestible carbohydrates of whole grains, promote production of short-chain fatty acids and may lead to enhanced glucose oxidation and insulin clearance.^(12,34)^ Soluble fiber and indigestible carbohydrates could also decrease intestinal transit time, delay gastric emptying and intestinal absorption and may consequently decrease glucose and insulin responses.^(34)^ High content of magnesium in DASH diet along with high fiber content may also improve insulin sensitivity.^(35)^ The high arginine content of DASH diet is also another explanation for its beneficial effect on IR and serum insulin levels; DASH diet contains high amounts fish, soy, beans, lentils, whole grains, and nuts, parsley, and fresh basil that enriched with arginine, and it has been proposed that arginine can improve IR by increasing production of nitric oxide and.^(36)^ DASH eating pattern is also rich in dietary calcium that mediate its effects on IR through effect on inflammatory pathways, calcitriol and gene expression.^(37)^ Anti-inflammatory effects of DASH diet on serum high-sensitive C-reactive protein levels may also explain its beneficial effect on insulin homoeostasis.^(29,32)^

Our findings should be interpreted considering to some pints; assessment of dietary intakes was at only one time point and the dietary patterns were assessed based on food intake data without considering eating behaviors such as meal and snacking pattern. Lack of data on postprandial levels of insulin to calculate the disposition index and accurately justify insulin homeostasis parameters, was a limitation for explanation of the observed associations. Regardless of these issues, to our knowledge, this is the first study investigated DASH diet score in relation to IR in a non-diabetes Asian population over the time. Use of a validated FFQ to assess regular dietary intake and determine major dietary pattern was an important strength of this study.

In conclusion, our findings showed that more adherence to DASH diet, identified as higher DASH score, decreased the risk of IR The principle implications of this study highlight the importance of diet in the development and prevention of disglycemia and impaired insulin homeostasis.

## Figures and Tables

**Table 1:** Characteristics of the participants across tertiles of DASH diet score

Characteristics	Dietary DASH score			*p* value
	T1: 9–20 (*n* = 358)	T2: 20–24 (*n* = 326)	T3: 24–35 (*n* = 234)	
Age (year)	37.84 ± 11.72	41.63 ± 12.19	41.96 ± 12.10	0.001
Men (%)	47	32.5	20.5	0.001
Current smoker (%)	12.6	12.9	8.6	0.24
Weight (kg)				
Baseline	71.92 ± 13.29	70.58 ± 12.85	71.04 ± 12.37	0.4
After 3 years	73.99 ± 13.04	72.32 ± 12.65	72.41 ± 12.84	0.18
Waist circumference (cm)				
Baseline	88.17 ± 12.28	88.15 ± 12.25	88.33 ± 12.20	0.98
After 3 years	92.32 ± 10.78	92.60 ± 11.09	92.86 ± 11.19	0.83
Serum Insulin (µIU/ml)				
Baseline	7.28 ± 3.05	7.80 ± 3.07	7.68 ± 2.98	0.07
After 3 years	8.81 ± 4.23	8.54 ± 4.13	8.49 ± 4.27	0.6
HOMA IR				
Baseline	1.56 ± 0.67	1.66 ± 0.66	1.67 ± 0.68	0.06
After 3 years	2.10 ± 1.13	2.06 ± 1.29	2.02 ± 1.19	0.76

DASH, Dietary Approaches to Stop Hypertension; BMI, body mass index; WC, waist circumference; HOMA-IR, Homeostasis Model of Assessment Insulin Resistance. *Significant difference (*p*<0.05) using χ^2^ for categorical independent variables and one way ANOVA for continuous independent variables. ^a^Means ± SD.

**Table 2:** Characteristics of the participants across tertiles of DASH, Mediterranean diet and Healthy Eating Score

Characteristic	MED score				HEI score			
	T1: 11–23 (*n* = 389)	T2: 24–26 (*n* = 278)	T3: 27–34 (*n* = 260)	*p* value	T1: 17–38 (*n* = 333)	T2: 39–46 (*n* = 293)	T3: 47–87 (*n* = 301)	*p* value
Age (year)	38.34 ± 11.94	40.19 ± 11.91	43.52 ± 12.05	<0.0001*	38.73 ± 11.50	40.21 ± 12.16	42.26 ± 12.56	0.001*
Men (%)	47.3	43.2	44.2	0.53	49.5	44.7	40.9	0.08
Current smoker (%)	10.8	6.7	6.5	0.1	14.4	12.1	7.9	0.055
Weight (kg)								
Baseline	71.10 ± 13.39	71.59 ± 13.34	70.92 ± 11.56	0.81	70.88 ± 12.46	71.42 ± 12.78	71.32 ± 13.47	0.85
After 3 years	72.89 ± 13.07	73.20 ± 13.21	72.80 ± 12.17	0.92	72.98 ± 12.51	72.92 ± 12.59	72.97 ± 13.53	0.99
Waist circumference (cm)								
Baseline	87.78 ± 12.65	88.33 ± 13.04	88.72 ± 10.63	0.62	87.87 ± 11.79	88.07 ± 12.92	88.72 ± 12.05	0.66
After 3 years	91.93 ± 11.22	92.80 ± 11.04	93.28 ± 10.61	0.29	92.13 ± 10.59	92.35 ± 11.37	92.27 ± 11.08	0.4
Serum insulin (µIU/ml)								
Baseline	7.48 ± 3.06	7.65 ± 2.93	7.63 ± 3.04	0.74	7.61 ± 3.07	7.56 ± 2.94	7.55 ± 3.12	0.96
After 3 years	8.57 ± 4.12	8.73 ± 4.39	8.59 ± 4.14	0.89	8.60 ± 4.17	8.86 ± 3.89	8.43 ± 4.52	0.45
HOMA IR								
Baseline	1.59 ± 0.67	1.65 ± 0.66	1.65 ± 0.68	0.49	1.62 ± 0.66	1.61 ± 0.65	1.65 ± 0.71	0.74
After 3 years	2.01 ± 1.10	2.10 ± 1.41	2.09 ± 1.12	0.55	2.01 ± 1.05	2.12 ± 1.16	2.06 ± 1.40	0.54

HEI, healthy eating index; MED, Mediterranean diet; HOMA-IR, Homeostasis Model of Assessment Insulin Resistance. *Significant difference (*p*<0.05) using χ^2^ for categorical independent variables and one way ANOVA for continuous independent variables. Means ± SD.

**Table 3:** Nutrients and Food group intake across tertiles of DASH diet score

Nutrients and food group	Dietary DASH score			*p* value
	T1: 9–20 (*n* = 358)	T2: 20–24 (*n* = 326)	T3: 24–35 (*n* = 234)	
Total energy (kcal/day)	2,390.70 ± 38.26^a^	2,121.44 ± 38.84	2,298.20 ± 43.31	0.001
Carbohydrate (% of energy)	57.36 ± 0.39	57.35 ± 0.39	58.26 ± 0.44	0.22
Protein (% of energy)	13.55 ± 0.13	13.57 ± 0.13	13.95 ± 0.15	0.09
Total Fat (% of energy)	30.97 ± 0.38	31.68 ± 0.39	31.26 ± 0.43	0.43
Saturated fat (% of energy)	10.37 ± 0.16	10.51 ± 0.16	10.45 ± 0.18	0.84
Monounsaturated fat (% of energy)	10.72 ± 0.15	11.01 ± 0.15	10.75 ± 0.18	0.35
Polyunsaturated fat (% of energy)	6.46 ± 0.12	6.71 ± 0.12	6.44 ± 0.14	0.27
Cholesterol (mg/day)	241.65 ± 7.10	206.42 ± 7.21	226.68 ± 8.04	0.002
Potassium (mg/day)	3,369.67 ± 73.44	3,502.35 ± 74.55	4,511.52 ± 83.13	0.001
Calcium (mg/day)	1,191.08 ± 27.18	1,167.32 ± 27.59	3,841.32 ± 30.76	0.001
Magnesium (mg/day)	375.86 ± 7.39	350.80 ± 7.50	405.37 ± 8.37	0.001
Fiber (g/day)	21.96 ± 0.56	23.96 ± 0.57	32.67 ± 0.63	0.001
Food group (serving/day)				
Vegetable	1.53 ± 0.08	2.20 ± 0.08	3.37 ± 0.09	0.001
Fruit	2.07 ± 0.12	3.71 ± 0.12	5.60 ± 0.14	0.001
Low fat dairy	0.61 ± 0.03	0.82 ± 0.76	1.04 ± 0.03	0.001
Grains	10.14 ± 0.19	8.30 ± 0.20	7.00 ± 0.23	0.001
Legume nut	1.64 ± 0.07	2.04 ± 0.08	2.29 ± 0.09	0.001
Red meat	0.46 ± 0.01	0.36 ± 0.01	0.31 ± 0.02	0.001
Sweetened beverage	0.18 ± 0.01	0.09 ± 0.01	0.06 ± 0.01	0.001

DASH, Dietary Approaches to Stop Hypertension. *Significant difference (*p*<0.05) using one way ANOVA. ^a^All values are means ± SE.

**Table 4:** Nutrients intake by tertile of DASH, Mediterranean diet and Healthy Eating Score

Nutrients	MED score				HEI score			
	T1: 11–23 (*n* = 389)	T2: 24–26 (*n* = 278)	T3: 27–34 (*n* = 260)	*p* value	T1: 17–38 (*n* = 333)	T2: 39–46 (*n* = 293)	T3: 47–87 (*n* = 301)	*p* value
Total energy (kcal/day)	2,603.63 ± 32.77	2,140.81 ± 38.76	1,907.10 ± 40.08	<0.0001	2,303.73 ± 38.93	2,296.07 ± 41.50	2,205.69 ± 40.94	0.16
CHO (% of energy)	55.29 ± 0.34	57.94 ± 0.41	60.73 ± 0.42	<0.0001	57.66 ± 0.39	57.42 ± 0.42	57.75 ± 0.41	0.84
Protein (% of energy)	13.66 ± 0.12	13.51 ± 0.14	13.79 ± 0.15	0.62	12.76 ± 0.12	13.63 ± 0.13	14.73 ± 0.13	<0.0001
Total Fat^a^ (% of energy)	33.26 ± 0.34	31.09 ± 0.40	28.60 ± 0.42	<0.0001	31.64 ± 0.38	31.55 ± 0.41	30.68 ± 0.40	0.17
SFA (% of energy)	11.15 ± 0.14	10.32 ± 0.17	9.51 ± 0.17	<0.0001	10.67 ± 0.16	10.33 ± 0.17	10.30 ± 0.17	0.19
MUFA (% of energy)	11.55 ± 0.13	10.73 ± 0.16	9.87 ± 0.16	<0.0001	10.96 ± 0.15	10.99 ± 0.16	10.54 ± 0.16	0.08
PUFA (% of energy)	6.99 ± 0.11	6.55 ± 0.13	5.88 ± 0.14	<0.0001	6.67 ± 0.12	6.74 ± 0.13	6.21 ± 0.13	0.009
Cholesterol (mg/dl)	268.64 ± 6.37	202.77 ± 7.54	183.37 ± 7.79	<0.0001	225.14 ± 7.19	226.49 ± 7.67	223.31 ± 7.56	0.95
K^+^ (mg)	3,976.51 ± 71.99	3,552.79 ± 85.16	3,587.84 ± 88.06	<0.0001	3,295.71 ± 76.08	3,818.42 ± 81.10	4,156.51 ± 80.02	<0.0001
Ca^2+^ (mg)	1,363.65 ± 25.14	1,161.39 ± 29.74	1,130.21 ± 30.75	<0.0001	1,159.33 ± 27.57	1,239.88 ± 29.39	1,321.73 ± 28.99	<0.0001
Mg^2+^ (mg)	407.16 ± 6.83	357.14 ± 8.08	347.37 ± 8.35	<0.0001	350.59 ± 7.45	380.49 ± 7.94	397.87 ± 7.84	<0.0001
Fiber (g)	25.79 ± 0.57	25.46 ± 0.67	25.85 ± 0.69	0.9	22.91 ± 0.60	26.11 ± 0.64	28.40 ± 0.63	<0.0001

HEI, healthy eating index; MED, Mediterranean diet; MUFA, monounsaturated fatty acids; CHO, carbohydrates; PUFA, polyunsaturated fatty acids; SFA, saturated fatty acids; K^+^, potassium; Ca^2+^, calcium; Mg^2+^, magnesium. *Significant difference (*p*<0.05) using one way ANOVA.

**Table 5:** Food group intake by tertile of Mediterranean diet score based on quintile scoring system for each component

Food group (g/day)	MED score			
	T1: 11–23 (*n* = 389)	T2: 24–26 (*n* = 278)	T3: 27–34 (*n* = 260)	*p* value
Vegetables	215.15 ± 9.15	280.78 ± 10.83	336.35 ± 11.20	<0.0001
Legumes	4.25 ± 0.92	9.82 ± 1.09	12.67 ± 1.13	<0.0001
Fruits and nuts	237.74 ± 13.44	317.89 ± 15.90	380.32 ± 16.45	<0.0001
Cereal	379.95 ± 9.31	399.62 ± 11.01	401.33 ± 11.38	0.24
Fish	4.43 ± 0.73	6.97 ± 0.87	9.93 ± 0.90	<0.0001
Meat	25.18 ± 1.07	17.21 ± 1.27	13.58 ± 1.31	<0.0001
Poultry	28.91 ± 1.40	19.00 ± 1.66	12.79 ± 1.71	<0.0001
Whole dairy	567.46 ± 3.90	512.28 ± 4.62	484.42 ± 4.77	<0.0001

MED, Mediterranean diet. *Significant difference (*p*<0.05) using one way ANOVA.

**Table 6:** Food group intake by tertile of HEI Score

Food groups	HEI score			
	T1: 17–38 (*n* = 333)	T2: 39–46 (*n* = 293)	T3: 47–87 (*n* = 301)	*p* value
Total fruit (cup)	0.80 ± 0.03	1.09 ± 0.04	1.38 ± 0.04	<0.0001
Whole fruit (cup)	0.78 ± 0.03	1.06 ± 0.04	1.35 ± 1.03	<0.0001
Total vegetables (cup)	0.99 ± 0.03	1.21 ± 0.03	1.44 ± 0.03	<0.0001
Greens and beans (cup)	0.14 ± 0.00	0.18 ± 0.01	0.25 ± 0.01	<0.0001
Whole grains (g/day)	6.75 ± 0.97	11.49 ± 1.04	19.09 ± 1.02	<0.0001
Dairy (cup)	0.79 ± 0.02	0.91 ± 0.02	1.07 ± 0.02	<0.0001
Total protein foods (g/day)	32.13 ± 1.06	37.19 ± 1.3	40.02 ± 1.12	<0.0001
Seafood and plant proteins (g/day)	8.23 ± 0.44	10.92 ± 0.47	14.41 ± 0.46	<0.0001
Fatty acid [(PUFAs + MUFAs)/SFAs] (g/day)	1.73 ± 0.02	1.78 ± 0.03	1.70 ± 0.30	0.2
Refined grains (g/day)	205.22 ± 3.80	178.47 ± 4.05	153.98 ± 3.99	<0.0001
Sodium (g/day)	2.59 ± 0.08	1.80 ± 0.08	1.60 ± 0.08	<0.0001
Empty calories (g/day)	134.17 ± 3.82	115.06 ± 4.07	71.96 ± 4.01	<0.0001

HEI, healthy eating index. *Significant difference (*p*<0.05) using one way ANOVA.

**Table 7:** Odds and 95% confidence interval for occurrence of the insulin resistance in each tertile categories of DASH, Mediterranean diet and Healthy Eating Score

	DASH				MED				HEI			
	T1: 9–20 (*n* = 358)	T2: 20–24 (*n* = 326)	T3: 24–35 (*n* = 234)	*p* trend	T1: 11–23 (*n* = 389)	T2: 24–26 (*n* = 278)	T3: 27–34 (*n* = 260)	*p* trend	T1: 17–38 (*n* = 333)	T2: 39–46 (*n* = 293)	T3: 47–87 (*n* = 301)	*p* trend
Model 1	1	0.89 (CI: 0.57–1.38)	0.60 (CI: 0.36–1.00)	0.058	1	0.99 (CI: 0.62–1.58)	1.07 (CI: 0.67–1.71)	0.77	1	1.06 (CI: 0.67–1.69)	0.91 (CI: 0.57–1.47)	0.68
Model 2	1	0.79 (CI: 0.50–1.24)	0.52 (CI: 0.31–0.89)	0.01	1	0.96 (CI: 0.60–1.53)	0.97 (CI: 0.60–1.55)	0.88	1	1.02 (CI: 0.64–1.63)	0.84 (CI: 0.52–1.36)	0.45
Model 3	1	0.76 (CI: 0.47–1.21)	0.46 (CI: 0.26–0.80)	0.007	1	0.85 (CI: 0.52–1.40)	1.04 (CI: 0.64–1.71)	0.96	1	0.99 (CI: 0.61–1.61)	0.80 (CI: 0.48–1.33)	0.38
Model 4	1	0.87 (CI: 0.52–1.45)	0.49 (CI: 0.26–0.90)	0.02	1	0.99 (CI: 0.58–1.168)	1.21 (CI: 0.71–2.07)	0.52	1	1.20 (CI: 0.71–2.04)	0.98 (CI: 0.57–1.70)	0.88
Model 5	1	0.80 (CI: 0.46–1.38)	0.39 (CI: 0.20–0.76)	0.007	1	0.96 (CI: 0.55–1.69)	1.07 (CI: 0.60–1.91)	0.84	1	1.18 (CI: 0.67–2.08)	0.86 (CI: 0.48–1.55)	0.56

HEI, healthy eating index; MED, Mediterranean diet; DASH, dietary approaches to stop hypertension. Model 1: unadjusted for confounders. Model 2: adjustment for age and gender. Model 3: additional adjustment for body mass index and WC. Model 4: additional adjustment for smoking and physical activity. Model 5: additional adjustment for HOMA IR and serum insulin at baseline and anti-diabetic medications.
